# Adaptive Modulation of Adult Brain Gray and White Matter to High Altitude: Structural MRI Studies

**DOI:** 10.1371/journal.pone.0068621

**Published:** 2013-07-16

**Authors:** Jiaxing Zhang, Haiyan Zhang, Jinqiang Li, Ji Chen, Qiaoqing Han, Jianzhong Lin, Tianhe Yang, Ming Fan

**Affiliations:** 1 Department of Physiology and Neurobiology, Medical College of Xiamen University, Xiamen, Fujian, China; 2 Department of Clinical Psychology, Gulangyu Sanatorium of PLA, Xiamen, Fujian, China; 3 Magnetic Resonance Center, Zhongshan Hospital, Medical College of Xiamen University, Xiamen, Fujian, China; 4 Department of Cognitive Sciences, Institute of Basic Medical Sciences, Beijing, China; University of Jaén, Spain

## Abstract

The aim of this study was to investigate brain structural alterations in adult immigrants who adapted to high altitude (HA). Voxel-based morphometry analysis of gray matter (GM) volumes, surface-based analysis of cortical thickness, and Tract-Based Spatial Statistics analysis of white matter fractional anisotropy (FA) based on MRI images were conducted on 16 adults (20–22 years) who immigrated to the Qinghai-Tibet Plateau (2300–4400 m) for 2 years. They had no chronic mountain sickness. Control group consisted of 16 matched sea level subjects. A battery of neuropsychological tests was also conducted. HA immigrants showed significantly decreased GM volumes in the right postcentral gyrus and right superior frontal gyrus, and increased GM volumes in the right middle frontal gyrus, right parahippocampal gyrus, right inferior and middle temporal gyri, bilateral inferior ventral pons, and right cerebellum crus1. While there was some divergence in the left hemisphere, surface-based patterns of GM changes in the right hemisphere resembled those seen for VBM analysis. FA changes were observed in multiple WM tracts. HA immigrants showed significant impairment in pulmonary function, increase in reaction time, and deficit in mental rotation. Parahippocampal and middle frontal GM volumes correlated with vital capacity. Superior frontal GM volume correlated with mental rotation and postcentral GM correlated with reaction time. Paracentral lobule and frontal FA correlated with mental rotation reaction time. There might be structural modifications occurred in the adult immigrants during adaptation to HA. The changes in GM may be related to impaired respiratory function and psychological deficits.

## Introduction

A large number of people move from lowlands to high-altitude (HA) each year due to work, study, or training, staying for several months to several years. For example, many people work at mines in South American Andes (4500 m) and at Mauna Kea Observatories on the Big Island of Hawaii (4200 m) [1]. Each October, United States Antarctic Program participants go in for summer work at the Amundsen-Scott South Pole Station (2835 m) [2]. In China, Qinghai-Tibet Railway (with an average altitude of 4500 m) was built by more than 100000 workers [3]. The underlying problem with HA is that there is less oxygen. With more and more people immigrating to HA, the effects of hypoxia on body have drawn more and more attention.

The cerebral effects of ascent to HA have been of persistent concern [4]–[8]. Macromorphological damages such as cerebral edema, cortical atrophy, and cortical and subcortical lesions have been found in a few individuals with acute mountain sickness [9]–[14], while microstructural alterations have been shown in mountain climbers after once or repeated exposure to extreme altitude [15], [16]. For immigrants who have been to HA for a long period of time, such as several months to several years, their peripheral physiological systems typically employ adaptive mechanisms such as alterations in respiratory and circulatory function, hemoglobin concentration, and arterial oxygen saturation [17]. Such alterations change oxygen transport in the cerebral blood flow, leading to cumulative changes in brain structure. Moreover, the brain is the control centre of the body. At HA, through afferent feedback, the adaptation in the cardiovascular and respiratory systems may act on the control centers in the brain. The brain structural changes in immigrant descendants who born and living at HA have been studied [18]. However, up till now, little is known about the adaptive responses of brain structure in adult immigrants who have lived in HA environments for several months to several years.

Functional neuroImaging studies have revealed that a large number of neocortical, cerebellar and brainstem regions were activated by dyspnoea [19]. These dyspnoea-activated brain regions have been shown to be impaired in patients with obstructive sleep apnea [20], [21] or chronic obstructive pulmonary disease [22] and in HA immigrant descendants [18]. The WM microstructural alterations have been shown in the corpus callosum, corticospinal tract, and cerebellum in HA immigrant descendants [18] and in climbers after a short-term single mountain climbing [15]. Therefore, we hypothesized that adult immigrants who have a long-standing HA exposure would have a similar cerebral responses.

In the present study, 16 sea level (SL) natives who have immigrated to the Qinghai-Tibet Plateau (2300–4400 m) for 2 years were recruited for this purpose. Quantitative analysis methods such as voxel-based morphometry (VBM), cortical surface based analysis (FreeSurfer), and Tract-Based Spatial Statistics (TBSS) based on MRI data were employed to measure gray matter (GM) and white matter (WM) microstructural changes. Recently, the preprocessing steps of VBM have been improved with the Diffeomorphic Anatomical Registration Through Exponentiated Lie algebra (DARTEL) registration method [23], which can achieve more accurate inter-subject registration of brain images. FreeSurfer was performed to verify VBM results. FreeSurfer is a freely available automated technique which allows quantification of cortical thickness and has a facility to label multiple brain regions [24]. TBSS is a recently introduced method, which uses diffusion tensor MR imaging (DTI) to measure differences in fractional anisotropy (FA) between groups. TBSS increases the sensitivity and the interpretability of the results compared with voxel-based approaches based purely on non-linear registration [25]. These methods have been used in our previous studies [18], [22].

Since deficits in short term memory, visual construction, procedural learning, and working memory tasks as well as increase in reaction time have been reported in mountain climbers [8], [26]–[29], in peoples living at moderate altitude for a long period of time [30], and in HA immigrant descendants [31], [32], we tested these cognitive functions in our study and expected to explore whether the regional brain changes could clarify the mechanisms. The effects of long term hypoxia on the central nervous system have been believed to be manifest as hypoxic ventilatory depression [33], [34], but it is not completely clear to date why and how this happens [35]. Therefore, in the present study, pulmonary function was also examined. We expected to elucidate the central structural mechanisms underlying them.

## Materials and Methods

### Subjects

Sixteen male healthy soldiers, who have garrisoned the frontiers in Qinghai-Tibet Plateau (2300–4400 m) for 2 years, were studied (Table 1; Table 2). They were originally native lowlanders born and have been living at SL (Fujian Province) before immigrating to HA. They had no chronic mountain sickness. Sixteen male control subjects, with comparable age, educational background, and race, were recruited in Xiamen (<100 m). They were native lowlanders from the same SL places (Fujian Province) as the HA immigrant soldiers and had left their hometown about 2 years. Among these controls, 4 subjects were mechanics, 3 subjects were biological laboratory technician, and 9 subjects were security guards. All subjects were right-handed, non-smokers, with normal body weight and body mass index, and had no documented neurological disorders or history of head injuries with loss of consciousness. The experimental protocol was approved by the Ethical Committee of Xiamen University. Before the experiments, the subjects were informed of the objectives, requirements and procedures of the experiments, and all subjects provided written informed consent forms, which were kept in archive. Subjects were compensated for participation. All potential participants who declined to participate or otherwise did not participate were eligible for treatment (if applicable) and were not disadvantaged in any other way by not participating in the study.

**Table 1:** Detailed information of immigrants living at HA.

Names of HA immigrants	The altitude and period of time of immigrants living in HA
Subject 1	2800 m: 1 month → 3600 m: 1 year and 11 months
Subject 2	2300 m: 2 years
Subject 3	2300 m: 2 years
Subject 4	4400 m: 2 years
Subject 5	3500 m: 2 years
Subject 6	3000 m: 2 years
Subject 7	4100 m: 2 years
Subject 8	3800 m: 2 years
Subject 9	4000 m: 2 years
Subject 10	4300 m : 2 years
Subject 11	3000 m: 1 year and 6 months → 2700 m: 6 months
Subject 12	3100 m: 2 years
Subject 13	3100 m: 2 years
Subject 14	2300 m: 6 months → 3600 m: 1 year and 6 months
Subject 15	2300 m: 2 years
Subject 16	4100 m: 2 years

**Table 2:** Demographic and physiological characteristics of subjects.

	HA immigrants	Controls	p
Number of subjects	16	16	
Age (years) (mean ± SD)	20.5±0.7 (20–22)	19.9±1.5 (17–22)	0.171
Weight	60.1±5.0 (53.0–71.2)	58.9±5.2 (54.6–65.0)	0.456
Education (years) (mean ± SD)	6.7±3.9 (3–12)	7.5±5.0 (3–14)	0.590
HGB (g/liter)	159.8±11.4 (144–189)	140.9±8.1 (127–154)	<0.001
RBC (10^12^/liter)	5.6±1.0 (4.4–7.8)	4.7±0.3 (4.2–5.3)	0.007
Pulmonary function testing			
VC (% predicted)	92.7±14.9 (67.8–119.3)	103.2±7.7 (92.9–120.4)	0.007
FVC (% predicted)	84.1±15.8 (49.6–103.4)	100.0±10.3 (78.6–117.5)	<0.001
FEV1 (% predicted)	78.2±14.6 (51.5–99.5)	96.5±15.5 (78.9–150.5)	<0.001
FEV25%	4.8±1.4 (2.4–6.4)	6.2±1.4 (3.3–8.4)	0.004
FEV50%	4.1±1.1 (2.4–5.9)	4.4±1.0 (2.2–5.6)	0.412
FEV75%	2.9±0.9 (1.4–3.9)	2.2±0.6 (1.2–4.2)	0.013

FEV, forced expiratory volume; FVC, forced vital capacity; HGB, hemoglobin; RBC, red blood cell; VC, vital capacity.

### Pulmonary Function and Neuropsychological Tests

Pulmonary function measure, neuropsychological tests, and MRI scans were conducted in Zhongshan Hospital, Xiamen, Fujian province, China, within seven days after HA immigrants gradually descended to sea level. The neuropsychological tests included the following items: (1) number search task, which is thought to tap visual processing [36]. (2) memory search, which is retrieval from long-term memory [37]. (3) mental rotation, which detects spatial orientation ability and spatial visualization. We employed a version of the Gong et al. [38]. (4) the visual reproduction and digit span tasks, which taken from the Chinese revised version of Wechsler Memory Scale [39], were used to measure visual memory and short-term working memory, respectively. (5) the Rey-Osterrieth Complex Figure (ROCF), which assesses the short- and long-term visual memory and visuoconstructional ability. (6) serial reaction time task is used to measure simple visuomotor implicit procedural learning. The procedures of the ROCF and serial reaction time task have been adopted in our previous study [30]. Mood tests used Self-Rating Anxiety Scale and Self-Rating Depression Scale. Independent t test and ANOVA analyzed between-group differences. Statistical significance was set at p<0.05.

### MRI Data Acquisition

Images were acquired on a Siemens Trio Tim 3.0T (Erlangen, Germany). A 3D structural MRI was acquired using a T1-weighted MPRAGE sequence (TR/TE = 1900 ms/2.7 ms, FOV = 25 × 25 cm^2^, NEX = 1, matrix = 256 × 246, slice thickness = 1.0 mm, and 176 sagittal slices in the third dimension. Conventional 2D T1 and T2 images were also acquired. A DTI pulse sequence with single shot diffusion-weighted echo planar imaging (TR/TE = 11000/96 ms, FOV = 184 × 184 mm^2^, NEX = 1, matrix = 128 × 128, slice thickness = 3 mm) was applied sequentially in 30 non-collinear directions (b-value = 1000 s/mm^2^) with one scan without diffusion weighting (b = 0 s/mm^2^). We acquired 50 contiguous slices covering the whole brain. The data analysis was conducted by two researchers who were blind to the status of subjects.

### VBM Analysis

The 3D T1 images were used for GM analysis using VBM8 toolbox implemented in SPM8 (Wellcome Department of Imaging Neuroscience, University College London, London, UK). Calculations and image matrix manipulations were performed using MATLAB (MathWorks, Natick, Massachusetts). The steps included: (i) the images were inspected and set at the anterior commissure. Each reorientated image was segmented into GM, WM, and cerebrospinal fluid in native space and Procrustes aligned GM images were generated by a rigid transformation. (ii) the DARTEL was used to create a study-specific template by the aligned images from all the subjects to improve inter-subject registration of structural images [23]. (iii) the normalized images were transformed into MNI space. These GM images were then smoothed using a Gaussian kernel of 8 mm full-width at half-maximum. Independent t-tests were performed to examine between-group differences, using age, education, and total intracranial volume as covariates. Levene’s test of equality of error variances was performed. The statistical parametric map was generated at t>3.7473, p<0.001 (uncorrected for multiple comparisons).

### Surface-based Analysis

Cortical thickness measurements were performed using the software FreeSurfer version 5.1.0 (http://surfer.nmr.mgh.harvard.edu/). The cortical surface was reconstructed using a semiautomated approach [40]. All registered MRIs were segmented to identify GM/WM boundaries. Thickness measurements were obtained by reconstructing representations of the GM/WM boundary and the white boundary to the GM/cerebrospinal fluid boundary and then calculating the closest distance from those surfaces at each vertex on the tessellated surfaces. All subjects’ data were resampled to the freeSurfer default common surface template using a high-resolution surface-based averaging technique that aligned cortical folding patterns. Finally, the surface data were spatial smoothed using a Gaussian kernel of 15 mm full-width at half-maximum. All images were carefully visually inspected to ensure accurate identification of the gray/white matter boundary and the pial surface. Regional cortical thickness variations were compared using general linear model at each vertex across the cortical surface, with cortical thickness as dependent variable and age, education, and total intracranial volume as covariates. The statistical parametric map was generated at p<0.05 and p<0.001 (uncorrected for multiple comparisons), respectively.

### TBSS Analysis

DCM2NII was used to convert diffusion tensor images from the proprietary scanner format to the NIFTI format. Then the images were processed using the FSL 4.1.5 software package (http://www.fmrib.ox.ac.uk/fsl/). Detailed processes were described in our previous studies [16], [18], [22]. TBSS processing includes the following steps: (i) align the FA images of all subjects to a template which was arbitrarily selected from those FA images by nonlinear registrations; (ii) transform all the aligned FA images into 1×1×1 mm^3^ MNI152 space by affine registrations to remove the effect of cross-subject spatial variability that remains after the non-linear registration; (iii) create the mean FA image and filter to retain only the centre of the WM tracts, with the threshold FA ≥0.20, and successfully exclude voxels, which consisted of GM or cerebrospinal fluid in the majority of subjects, so as to create the mean FA skeleton. (iv) project individual subjects’ FAs onto mean FA skeleton. (v) following these steps, data was fed into voxel-wise cross-subject statistical analyses. In all cases, the null distribution was built up over 5000 permutations. The groups were compared by ANCOVA using age and education as covariates. Levene’s test of equality of error variances was performed. The statistical parametric map was generated at p<0.05 (false discovery rate (FDR) corrected for multiple comparisons).

Within the cluster of changed FA, mean λ1 and λ23 values were extracted from each individual’s λ1 and λ23 maps. Independent t-tests were used to identify the group differences for these distinct brain locations. Statistical significance was set at p<0.05.

### Correlation Analyses of Brain Structures with Physiological/Neuropsychological Variables

GM, cortical thickness, and FA values in the changed regions were extracted from individual’s normalized and smoothed map. Then partial correlation was used to assess the correlations of GM volume, cortical thickness, and FA value with altitude, pulmonary variables, and neuropsychological measurements, controlling for age and education. Statistical significance was set at p<0.05.

## Results

### Physiological and Behavioral Findings

Compared with the controls, HA immigrants had significantly lower values in vital capacity (VC), forced vital capacity (FVC) as well as forced expiratory volume (FEV) in one second, 25%, and 75% (Table 2). No significant differences between HA immgrants and SL controls in anxiety (HA immigrants vs. SL controls: 41.67±9.59 vs. 40.33±9.32, p = 0.702) and depression (42.69±15.05 vs. 38.00±7.53, p = 0.266) scores. The test scores of the behaviors are shown in Table 3. Compared with controls, HA immigrants had increased reaction time in number search, memory search, and mental rotation tasks. Moreover, HA immigrants had lower score in the standard score of mental rotation test. There were no significant differences between groups in digit span task, visual reproduction, and ROCF task. In serial reaction time task, two-way ANOVA with repeated measures detected a significant increase in reaction time in HA immigrants relative to SL group (F_(1,20)_ = 8.50, p = 0.009).

**Table 3:** Behavioral results of HA immigrants and SL controls.

Tests	HA immigrants	SL controls	p
Number search			
Standard score	4.8±1.6	4.1±2.7	0.324
Reaction time (ms)	6730.9±648.3	6225.7±418.5	0.029
Memory search			
Standard score	7.0±0.8	6.2±1.5	0.064
Reaction time (ms)	6300.9±430.3	5616.3±425.2	<0.001
Mental rotation			
Standard score	1.3±0.9	2.9±2.8	0.045
Reaction time (ms)	15038.9±4181.0	8654.3±684.3	<0.001
Speed estimate	6.3±2.9	6.2±2.2	0.929
Digit span			
Forward task	8.7±1.2	8.9±1.1	0.756
Backward task	6.7±1.5	6.5±1.3	0.713
Visual reproduction	13.2±1.8	13.9±0.3	0.145
Rey-Osterrieth Complex Figure			
Immediate recall	26.0±6.3	27.3±6.9	0.642
Delayed recall	25.8±6.8	28.0±6.4	0.419

### GM Volume

No subject from either group showed visible abnormalities on T1-weighted structural images. There were no significant differences in the total volumes of GM (HA immigrants vs. SL controls: 641.1±45.6 vs. 660.8±43.5, p = 0.545), WM (531.4±33.6 vs. 556.2±55.0, p = 0.143), and cerebrospinal fluid (220.2±18.5 vs. 221.0±21.2, p = 0.905) between the two groups.

**Figure 1:**
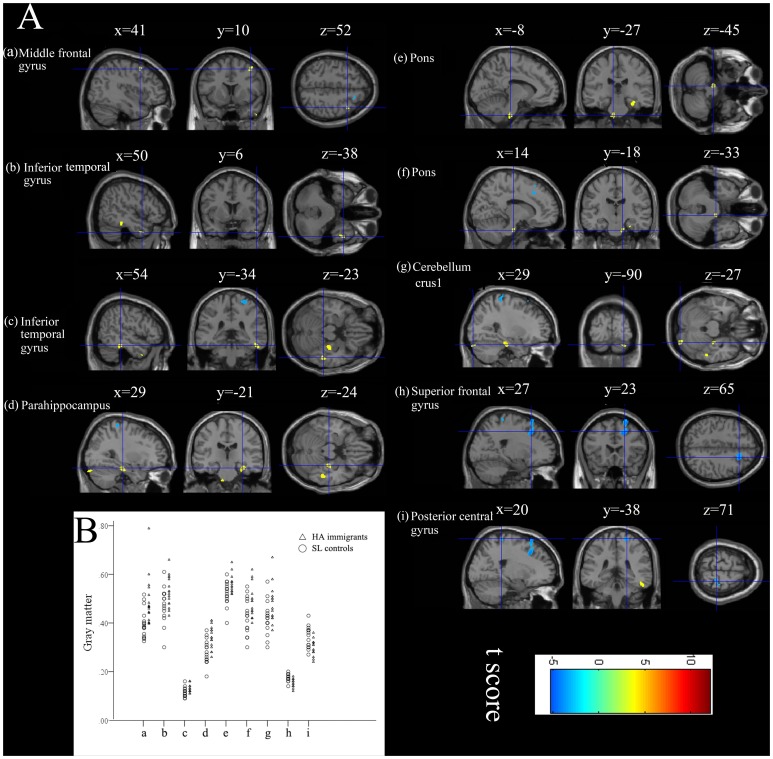
Changed gray matter volumes in HA immigrants compared with SL controls as revealed by voxel-based morphometry. (A) Three-dimensional slices depicting regions showing increased gray matter volume in the bilateral pons, and right cerebellum crus1 and decreased gray matter volume in right superior frontal gyrus and right posterior central gyrus overlaid on a T1-weighted MRI anatomical image in the stereotactic space of the MNI template. (B) Scatter plots show between-group changes in these clusters.

Compared with controls, HA immigrants had significantly decreased regional GM volumes in the right postcentral gyrus and right superior frontal gyrus; HA immigrants had significantly increased regional GM volumes in the right middle frontal gyrus, right parahippocampal gyrus, right inferior and middle temporal gyri, bilateral inferior ventral pons, and right cerebellum crus1 (Fig. 1; Table 4).

**Table 4:** Regional information of changed gray matter volume in HA immigrants compared with SL controls.

Areas	Volume (mm^3^)	Brodmann areas	MNI coordinate			t-score (peak)
			x	y	z	
Postcentral_R	166	3	20	−38	71	3.719
Frontal_Sup_R	640	8	27	23	65	5.089
Frontal_Mid_R	89	6	41	10	52	3.424
ParaHippocampal_R	199	36	29	−21	−24	4.426
Temporal_Inf_R	136	20	54	−34	−23	4.191
Temporal_Inf_R	64	21	50	6	−38	3.423
Pons_L	147		−8	−27	−45	4.187
Pons_R	63		14	−18	−33	3.972
Cerebellum_crus1_R	130		29	−90	−27	3.612

### Cortical Thickness

**Figure 2:**
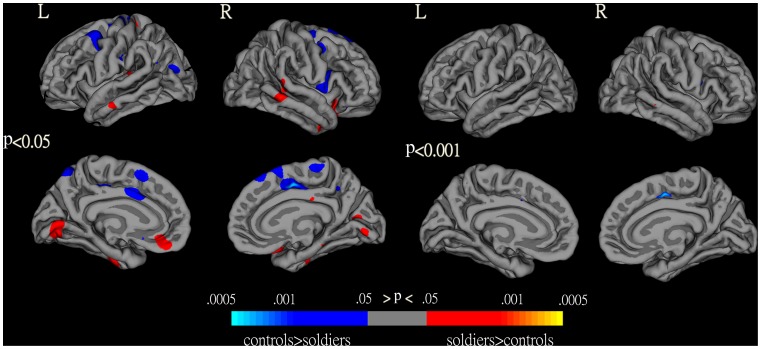
Changed cortical thickness in HA immigrants compared with SL controls as revealed by surface-based analysis. Maps are presented on the pial cortical surface.

HA immigrants had significantly changed cortical thickness in a broad range of brain areas compared with controls (p<0.05, uncorrected for multiple comparisons) (Fig. 2). The significantly decreased cortical thickness regions included the bilateral superior frontal gyri, bilateral caudal middle frontal gyri, bilateral precentral cortex, bilateral paracentral gyrus, right lateral orbitofrontal cortex, right pars opercularis, right precuneus, left posterior cingulate gyrus, left superior parietal cortex, and left inferior parietal cortex; The significantly increased cortical thickness regions included the right lateral orbitofrontal cortex, right middle temporal gyrus, right pericalcarine cortex, bilateral inferior temporal gyrus, left lingual gyrus, and left medial orbitofrontal cortex. However, when statistical significance was set at p<0.001 (uncorrected for multiple comparisons), only the right superior frontal gyrus showed significantly decreased cortical thickness (Fig. 2).

**Figure 3:**
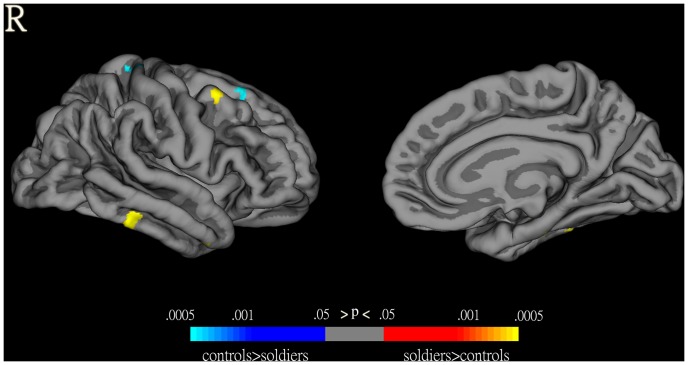
VBM-identified differences in gray matter volume projected on FreeSurfer-derived cortical surface. These maps show significant gray matter volume differences between HA immigrants and SL controls (compare these with the FreeSurfer analyses in Figure 2).

In addition, VBM-identified differences in gray matter volume were projected on FreeSurfer-derived cortical surface. Post-hoc analysis of thickness in the clusters of VBM findings showed significantly increased cortical thickness in the right inferior temporal gyrus (p = 0.032) and significantly decreased cortical thickness in the right superior frontal gyrus (p = 0.047) in HA immigrants compared with controls, which concurred with the cortical thickness findings (Fig. 3).

### FA, Longitudinal Diffusivity, and Radial Diffusivity

**Figure 4:**
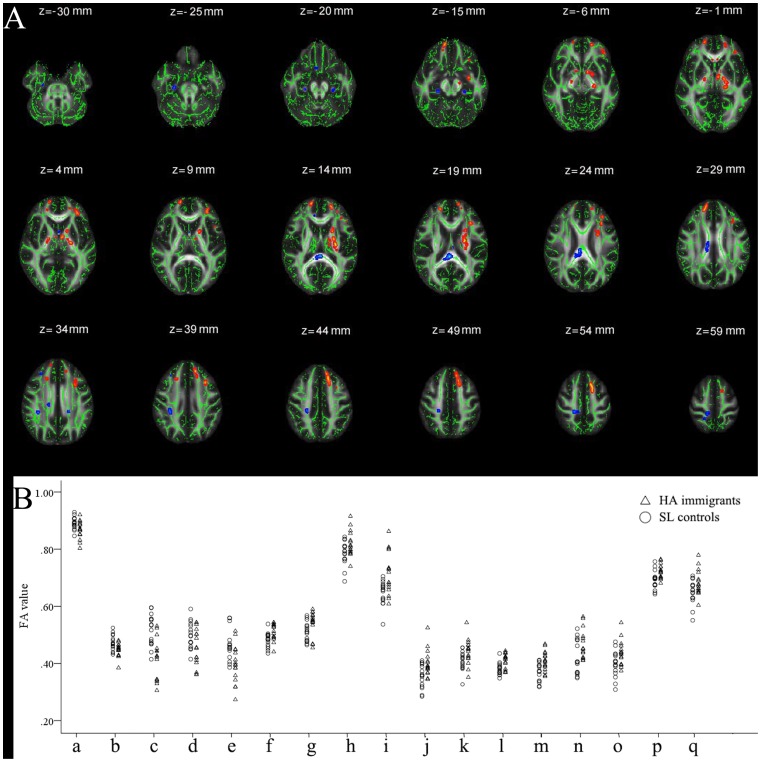
Regional changes in FA in HA immigrants compared with SL controls. (A) The group’s mean FA skeleton (green) was overlaid on the mean FA images. The threshold of mean FA skeleton was set at 0.2. (B) Scatter plots show between-group changes in these clusters.

Compared with SL controls, HA immigrants had significantly lower FA values in the forceps major of corpus callosum, right superior corona radiata (corresponding to paracentral lobule), right superior longitudinal fasciculus (corresponding to frontal lobe), and bilateral hippocampus; HA immigrants had significantly higher FA in the superior longitudinal fasciculus (corresponding to left superior and middle frontal gyrus and right superior frontal gyrus), right inferior longitudinal fasciculus (corresponding to inferior temporal gyrus), forceps minor of corpus callosum, left corticospinal tract, and left corticonuclear tract (Fig. 4, Table 5).

**Table 5:** Main regions showing greater and reduced FA in HA immigrants and SL controls.

Region Number	MNI (peak)			Voxels (mm^3^)	Tract	Corresponding cortical area	FA value		λ1 (×10^3^ mm^2^/s)		λ23 (×10^3^ mm^2^/s)	
	x	y	z				HA	Control	HA	Control	HA	Control
**HA<SL**												
(a)	4	−34	18	148	CC	Forceps major	0.869 (0.028)	0.894(0.029)	1.128(0.045)	1.168(0.087)	0.511(0.027)	0.475(0.031)*
(b)	16	−32	53	104	SCR-R	Paracentrallobule	0.456 (0.098)	0.464 (0.071)	1.714(0.055)	1.676(0.076)	0.202(0.042)	0.155(0.054)*
(c)	31	−33	38	46	SLF-R	Frontal lobe	0.462 (0.035)	0.522 (0.033)	1.096(0.105)	1.099(0.083)	0.955(0.242)	0.891(0.215)*
(d)	−23	−29	−16	20		Lefthippocampus	0.453 (0.044)	0.481 (0.022)	1.040(0.038)	1.101(0.042)	0.494(0.030)	0.464(0.030)*
(e)	26	−20	−25	17		Right hippocampus	0.455 (0.031)	0.468 (0.059)	1.196(0.062)	1.250(0.049)	0.561(0.061)	0.551(0.024)*
**HA>SL**												
(f)	−13	35	38	254	SLF-L	Superior frontal gyrus	0.542 (0.039)	0.526 (0.030)	1.168(0.038)	1.061(0.038)	0.410(0.038)	0.471(0.030)*
(g)	−14	7	54	112	SLF-L	Superior frontal gyrus	0.589 (0.057)	0.516 (0.042)	1.236(0.061)	1.267(0.100)	0.399(0.035)	0.649(0.057)*
(h)	−31	38	4	95	SLF-L	Superior frontal gyrus	0.447 (0.048)	0.415 (0.074)	1.076(0.082)	1.064(0.055)	0.567 (0.038)	0.570(0.038)*
(i)	−33	21	22	61	SLF-L	Middle frontal gyrus	0.483 (0.053)	0.451 (0.055)	1.052(0.044)	1.023(0.055)	0.557(0.057)	0.583(0.043)*
(j)	11	56	−10	52	SLF-R	Superior frontal gyrus	0.377 (0.076)	0.359 (0.063)	1.291(0.063)	1.221(0.036)	0.306(0.034)	0.361(0.031)*
(k)	−30	−5	−13	40	ILF-L	Inferior temporal gyrus	0.422 (0.043)	0.404 (0.051)	0.994(0.042)	0.973(0.034)	0.496(0.053)	0.513(0.037)*
(l)	13	53	27	79	CC	Forceps minor	0.445 (0.065)	0.373 (0.054)	1.021(0.067)	0.995(0.051)	0.550(0.045)	0.596(0.045)*
(m)	15	57	5	68	CC	Forceps minor	0.425 (0.05)	0.370 (0.043)	1.034(0.071)	1.062(0.071)	0.532(0.048)	0.572(0.055)*
(n)	−21	−8	18	57	CCT-L	Anterior limb of internal capsule	0.559 (0.092)	0.500 (0.059)	1.103(0.127)	1.078(0.011)	0.482(0.104)	0.519(0.009)*
(o)	−5	−6	−2	49	CNT-L	Posterior limb of internal capsule	0.733 (0.016)	0.675 (0.017)	1.164(0.093)	1.199(0.119)	0.598(0.075)	0.631(0.093)*
(p)	−26	−15	17	35	CCT-L	Posterior limb of internal capsule	0.727 (0.043)	0.663 (0.034)	1.049(0.045)	0.963(0.040)	0.521(0.061)	0.563(0.042)
(q)	−19	7	13	31	CCT-L	Posterior limb of internal capsule	0.705 (0.041)	0.635 (0.042)	1.400(0.034)	1.284(0.025)	0.324(0.018)	0.363(0.022)

CC, corpus callosum; CCT, corticospinal tract; CNT, corticonuclear tract; ILF, inferior longitudinal fasciculus; SCR, superior corona radiata; SLF, superior longitudinal fasciculus. Data are presented as means (SD). * p<0.05.

Lower FA values were associated with increased radial diffusivity and no changes of longitudinal diffusivity, while higher FA values were associated with decreased radial diffusivity and no changes of longitudinal diffusivity in HA immigrants vs. controls (Table 5).

### Correlations

**Figure 5:**
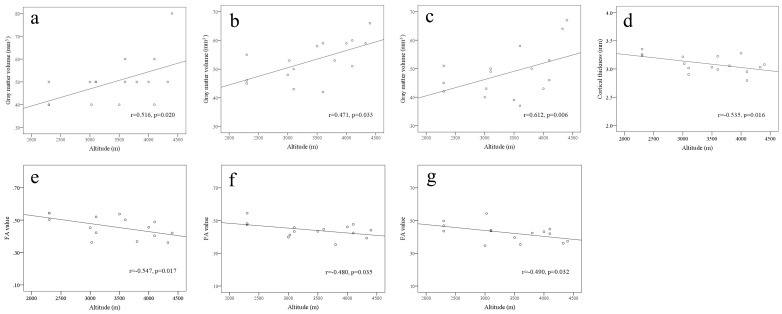
Correlations of gray matter volume, cortical thickness, and FA value with altitude in HA immigrants. Gray matter volumes in the left pons (a), right middle temporal gyrus (b), and right middle frontal gyrus (c); Cortical thickness values in the left superiorfrontal gyrus (d); FA values in the right superior longitudinal fasciculus (corresponding to superior frontal gyrus) (e), corpus callosum (forceps minor) (f), and middle frontal gyrus (g).

In HA immigrants, GM volumes in the left pons, right middle temporal gyrus, and right middle frontal gyrus had positive correlations with altitudes (Fig. 5 a, b, and c); Cortical thickness values in the left superiorfrontal gyrus had a negative correlation with altitude (Fig. 5 d); FA values in the right superior longitudinal fasciculus (corresponding to superior frontal gyrus), corpus callosum (forceps minor), and middle frontal gyrus had negative correlations with altitudes (Fig. 5 e, f and g).

**Figure 6:**
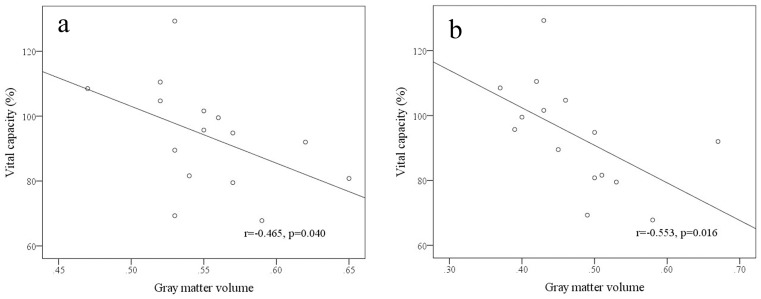
Correlation of gray matter volume with vital capacity value in HA immigrants. Gray matter volumes in the parahippocampal gyrus (a) and middle frontal gyrus (b).

In HA immigrants, GM volumes in the parahippocampal gyrus and middle frontal gyrus had significantly negative correlations with vital capacity (Fig. 6).

**Figure 7:**
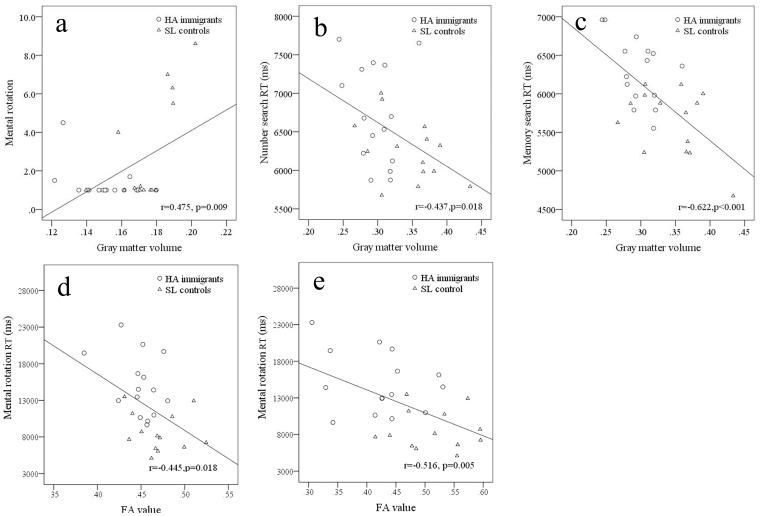
Correlations of gray matter volume and FA value with neuropsychological results. Gray matter volumes in the superior frontal gyrus (a) and postcentral gyrus (b, c). FA values in the right superior corona radiata (corresponding to paracentral lobule) (d) and right superior longitudinal fasciculus (corresponding to frontal lobe) (e).

In all subjects, GM volume in the superior frontal gyrus had significantly positive correlation with mental rotation and GM volume in the postcentral gyrus had significantly negative correlation with number search reaction time and memory reaction time, respectively (Fig. 7). In all subjects, FA value in the right superior corona radiata (corresponding to paracentral lobule) and right superior longitudinal fasciculus (corresponding to frontal lobe) had negative correlations with mental rotation reaction time (Fig. 7).

## Discussion

In the present study, we revealed that cerebral adaption in adult lowland natives who had spent two years living at HA was associated with brain structural modification, showing the changes of GM volumes and cortical thickness in a number of cortical regions, accompanied by the changes of anisotropy and diffusivity values in multiple sites of WM tracts. Moreover, increased GM volumes, cortical thickness, and FA in some regions in HA immigrants exhibited positive or negative correlations with altitudes. HA immigrants showed impairment in pulmonary function, deficits in mental rotation, and increase in reaction time. GM volumes and FA values in some changed regions had significant correlations with these physiological and psychological functions.

In the present study, the most consistent findings of VBM analysis and surface-based analysis were the changes in the right brain. Differences have also been detected, such as changed cortical thickness in the left brain revealed only by surface-based analysis. Differences between VBM and surface-based analyses may result from a number of steps in the data pre-processing. Surface-based analysis and VBM are alternative technique for identifying morphometric differences. However, VBM hinges on a number of assumptions, particularly the accuracy of image co-registration [41], [42]. VBM employs a nonlinear whole-brain registration, while surface-based uses a surface based registration of the cortical surface to a sphere [43]. Smoothing also differs between the two techniques, as in VBM, it is applied in three-dimensions, whereas for surface-based the smoothing is across the cortical surface [43]. In summary, VBM provides a mixed measure of cortical GM, including cortical surface area and/or cortical folding as well as cortical thickness; in contrast, surface-based analysis has the advantage of providing a quantitative value that represents a physical property of the cortical mantle [44].

The regions shown changes in GM in our study have been found activated by dyspnoea in a number of functional neuroimaging studies [19]. These dyspnoea-activated brain regions have been shown to be impaired in hypoxic patients. For example, the loss of GM in the superior frontal gyri, postcentral gyrus, inferior temporal gyrus, parahippocampal gyrus, quadrangular lobule (crus1) in the cerebellum were found in patients with obstructive sleep apnea [20], [21]. The loss of GM in the precentral gyrus and multiple sites in the temporal lobe were found in patients with chronic obstructive pulmonary disease in our previous study [22]. However, only a few regions, such as precentral cortex and parietal cortex, have been reported to be impaired in young HA immigrant descendants born and raising at HA [18] and in adult climbers who spend several times a year during 10 years in mountain climbing [15]. Moreover, no significant changes in regional GM were detected in adult climbers after mountain climbing one time [15]. In contrast, using VBM analysis method, the increases of GM volumes in several brain regions were found in patients with obstructive sleep apnea [45] and in patients with different types of dystonia, which is associated with hypoxia [46]. Taken together, these studies suggest a different response pattern in regional GM between various types of HA exposure.

In our study, WM FA values in corticospinal tract and corpus callosum were changed, which were consistent with previous findings in HA exposed populations and in hypoxic patients, suggesting corticospinal tract and corpus callosum are particularly susceptible to hypoxia. For example, the decrease of WM volume in the corticospinal tract was measured in repeated mountain climbers [15] and in patients with obstructive sleep apnea [47]. The decreases of WM FA in the corticospinal tract and corpus callosum were found in mountaineers after once mountain climbing [15], while the increases of FA in the corticospinal tract and corpus callosum were found in HA immigrant descendants [18].

Similar to our findings in brain structure, different patterns of cerebral blood supply and metabolism changes during acute and adaptive HA exposures are also occurred. Acute HA exposure increases cerebral blood flow markedly. However, if HA exposure lasts from more than 1 week to even several months or years, cerebral blood flow returns towards normal SL values. Cerebral blood flow in HA natives is usually even lower than that in lowlanders [48]. Cerebral metabolic rate of oxygen stays unchanged during acute HA exposure [49]. However, it decreases in HA residents [50].

According to Zatorre et al. [51], candidate mechanisms for adult GM increases may be related to neurogenesis, gliogenesis, synaptogenesis, and/or vascular changes. Whereas GM decreases may be resulted from anaerobic metabolic byproducts produced by hypoxia and from an increased release of glutamine from glutamatergic neurons following hypoxic exposure [29]. Here we suspected the increased GM during prolonged hypoxic exposure could be the result of at least one of the following: (1) Neurogenesis directly induced by hypoxia. Adult neocortex such as prefrontal, inferior temporal, and posterior parietal cortex have the capability of neurogenesis [52] and this neurogenesis can be induced [53]. In our previous studies, hypoxia has been proved to induce adult nurogenesis [54], [55] (2) Neurogenesis induced by afferent feedback (function-activated effects). The brain is the source of behavior, but in turn it is modified by the behaviors it produces. An example is that the increases of gray and white matter occurred with learning [51]. In the present study, spirometry changed and showed correlations with the increased GM in middle frontal premotor cortex and parahippocampal gyrus, which were activated by inspiratory and expiratory loads tasks [56]–[60], voluntary cough, sniff, and breathing [61]. (3) Gliogenesis. Global ischemia can activate microglia and macrophages proliferate [62]. (4) Angiogenesis. Prolonged hypoxia was observed to induce an increase in brain capillary density [63].

Our present study showed that the ventilatory functions of VC, FVC, and FEV1 at HA were significant decreased, which were consistent with the previous findings [33], [34]. Functional neuroimaging has demonstrated the sensorimotor cortex, cerebellum, supplementary motor and premotor areas involved in respiratory control and respiratory perception [64]. In the present study, the GM volumes changed in these regions, and among which the GM volumes in the parahippocampal gyrus and middle frontal gyrus had negative correlations with the vital capacity. The cortex is generally considered to act to inhibit the diencephalic areas that facilitate respiration. A number of studies on animals showed that stimulation of these cortical regions reduced ventilation, while decortication augmented ventilation [34]. Therefore, we suggest that hypoxia could stimulate these higher brain centers, resulted in neuronal cells increased, and in turn acted to depress respiration.

In this study, HA immigrants showed increased reaction times during various complex tasks, which agreed with the literature on the effects of altitude [29] and have also been found in HA immigrant descendants during performance of spatial and verbal working memory tasks [31], [32]. In the present study, the GM volume in right postcentral cortex had positive correlations with the number search reaction time and the memory search reaction time, indicating that the decreased GM volume in this region may be responsible for the increase in reaction time. This was supported by previous studies. For example, positron emission tomography study confirmed superior parietal locations associated with shift of attention [65]. The influence of a longer reaction time effect of memory search task was strong in a few cortices included the superior parietal regions [66]. In our study, HA immigrants showed impaired ability in performing mental rotation task. The decrease of GM volume in the superior frontal gyrus and lower FA in the right superior longitudinal fasciculus within frontal lobe may be responsible for this deficit, since GM volume in the superior frontal gyrus showed a positive correlation with mental rotation and right superior longitudinal fasciculus had negative correlations with mental rotation reaction time. Previous studies have confirmed the involvement of this region in mental rotation task [67]–[69].

There were several limitations in our present study. The first was that HA immigrants living at HA will be challenged in their emotional well-being such as being far away from dense crowds. HA immigrants will also be challenged by cultural change. Diet did not appear to be a factor in this change, because food similar to that in SL was available to the subjects. The second was that VBM has limitations. The VBM analysis investigated changes in GM distribution in the whole brain. The accuracy of VBM largely depends on the quality of the MR images, which rely on increasing the resolution of scans and the use of advanced pulse sequences in order to additionally detect qualitative changes in the cortex [70]. In the present study, FreeSurfer was performed to verify VBM results. This surface-based analysis revealed right hemispheric patterns of structural brain changes consistent with our VBM findings. In future studies, tensor-based morphometry (TBM) should be used to identify the highest accuracy for differentiating HA immigrants from SL controls. The strength of TBM is that it allows the measurement of the regional size and shape differences of sub-cortical structures for which one-to-one mappings are more clearly defined. VBM data are only registered to one template, while TBM works as a multi-template, addressing a higher possibility of misregistration [71]. The third was that it was not a self-control design.

In summary, our present study demonstrated regional GM and WM alterations in adult immigrants who have lived in HA for 2 years. Regional GM increases could be related to neurogenesis, gliogenesis, and/or angiogenesis, but the exactly mechanisms need to be further explored. Our findings, taken together with previous studies, suggest a different structural change between acute and prolonged HA exposure and a different adaptive response to HA between developmental and adult brain. Our finding may clarify the central mechanism of impairment in respiratory function. This study also revealed that HA adaptation occurred at the cost of deficits in some psychological performances. These deficits might be attributed to regional GM loss. Future research is needed to explore whether brain changes recover to normal after a return to sea level.
